# Posterior mediastinal Müllerian cyst in a patient with Mayer-Rokitansky-Küster-Hauser syndrome: a case report

**DOI:** 10.1093/jscr/rjag609

**Published:** 2026-07-22

**Authors:** Oleg Aleksandrov, Davide Patrini, Mario De Luca, David Moore, Andreas Gkikas, Amali Jayathilaka

**Affiliations:** Department of Thoracic Surgery, University College London Hospitals NHS Foundation Trust, 250 Euston Road, London NW1 2PG, United Kingdom; Department of Thoracic Surgery, University College London Hospitals NHS Foundation Trust, 250 Euston Road, London NW1 2PG, United Kingdom; Department of Thoracic Surgery, University College London Hospitals NHS Foundation Trust, 250 Euston Road, London NW1 2PG, United Kingdom; Department of Thoracic Surgery, University College London Hospitals NHS Foundation Trust, 250 Euston Road, London NW1 2PG, United Kingdom; Department of Thoracic Surgery, University College London Hospitals NHS Foundation Trust, 250 Euston Road, London NW1 2PG, United Kingdom; Department of Thoracic Surgery, University College London Hospitals NHS Foundation Trust, 250 Euston Road, London NW1 2PG, United Kingdom

**Keywords:** mediastinal cyst, Mayer–Rokitansky–Küster–Hauser syndrome, thoracic surgery, VATS, Müllerian cyst, posterior mediastinum

## Abstract

Posterior mediastinal cysts are rare lesions accounting for ~5%–10% of mediastinal cysts. Most are bronchogenic, enteric, or neurogenic in origin; however, Müllerian cysts have recently been recognized as a distinct entity thought to arise from displaced Müllerian duct tissue. Mayer-Rokitansky-Küster-Hauser (MRKH) syndrome results from incomplete development of Müllerian ducts and is characterized by congenital absence of the uterus and upper vagina. A shared embryological origin suggests a possible association between MRKH syndrome and mediastinal Müllerian cysts. We report the first known case of a posterior mediastinal cyst in a patient with MRKH syndrome type II treated successfully with uniportal video-assisted thoracoscopic surgery. We discuss the clinical presentation, histopathological findings, surgical management, and potential embryological link between these conditions.

## Introduction

Mayer-Rokitansky-Küster-Hauser (MRKH) syndrome is a rare congenital condition affecting ~1 in 4500 females, defined by aplasia or hypoplasia of the uterus and upper vagina due to abnormal development of the Müllerian ducts [1]. It is classified into type 1 (isolated genital anomalies) and type 2 (with extragenital malformations, such as renal or skeletal abnormalities) [2]. Mediastinal cysts are uncommon, comprising 12%–30% of mediastinal masses, and typically include bronchogenic, thymic, or enteric types [3]. Rare cases of mediastinal cysts with Müllerian differentiation, known as Hattori cysts, have been reported in the posterior mediastinum, often in perimenopausal women [4]. This report presents a case of a posterior mediastinal cyst in a patient with MRKH syndrome type 2, treated surgically, and discusses a possible embryological association.

## Case report

A 41-year-old female presented with left-sided chest pain radiating to the scapula, accompanied by numbness and weakness in the left arm, night sweats, and fatigue.

Magnetic resonance imaging (MRI) of the spine revealed a left posterior mediastinal cyst at T4–T5, causing pressure on vertebral bodies without spinal canal extension. Minor degenerative changes in the lumbar and cervical spine were noted, unlikely to explain symptoms. Contrast-enhanced computed tomography (CT) of the thorax confirmed a smooth, non-enhancing cystic lesion in the left paravertebral recess at T4–T6, measuring 15 × 20 × 38 mm with a density of 22 Hounsfield units, conforming to adjacent structures without capsule, calcification, or invasion (Fig. 1).

**Figure 1:**
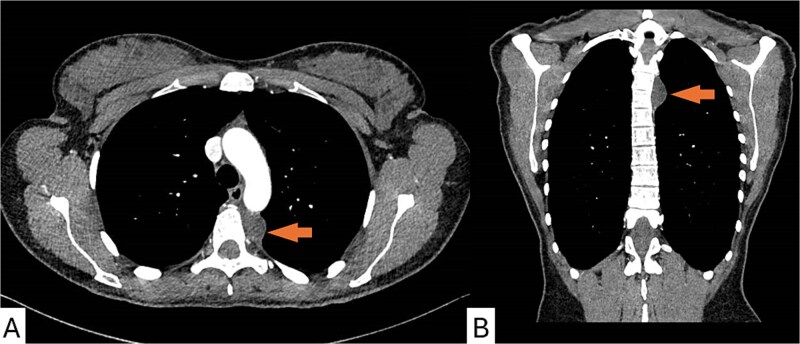
Contrast-enhanced CT scan of the chest demonstrating the posterior mediastinal cyst. (A) Axial view showing a well-defined, low-attenuation cystic lesion in the left posterior mediastinum at the T4–T5 level. (B) Coronal (frontal) view illustrating the lesion’s position in the paravertebral space, with no evidence of surrounding invasion.

Her medical history included MRKH syndrome type 2, characterized by primary amenorrhoea, absent uterus, short vagina (5 cm), recurrent urinary tract infections, left renal agenesis with compensatory hypertrophy of the right kidney, bilateral glaucoma, and chronic back pain managed with codeine. Genetic testing revealed a normal karyotype. The case was discussed at a multidisciplinary team meeting, and surgical intervention was recommended.

The patient underwent left uniportal VATS removal of the cyst. The cyst was aspirated, yielding serous fluid sent for microbiology, culture, sensitivity, and cytology. Histopathology showed a fibrous cyst wall lined by bland mesothelial cells, without inflammation or malignancy. Immunohistochemistry was positive for calretinin and WT1 (Fig. 2). Postoperative recovery was uncomplicated; the chest drain was removed on Day 1 after confirming no air leak and minimal output. The patient was reassured regarding benign histology. At the 9-month post-operative follow-up, clinical and radiological evaluations demonstrated no evidence of disease recurrence.

**Figure 2:**
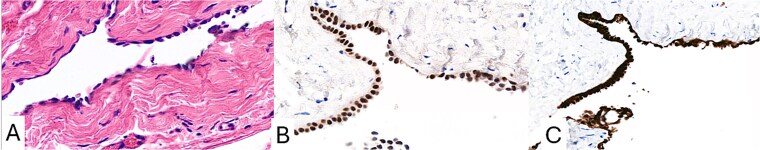
Histological and immunohistochemical features of the mediastinal cyst. (A) Haematoxylin and eosin (H&E) stain showing a fibrous cyst wall lined by a single layer of bland mesothelial cells. (B) Immunohistochemical staining demonstrating diffuse nuclear positivity for WT1 in the cyst lining. (C) Immunohistochemical staining showing strong cytoplasmic positivity for calretinin.

## Discussion

Mediastinal cysts are typically benign and asymptomatic but may cause symptoms through compression, including chest pain and neurological symptoms. The occurrence of a mediastinal cyst in a patient with MRKH syndrome raises the possibility of a Müllerian origin. MRKH syndrome results from disrupted Müllerian duct development during embryogenesis, where the ducts, derived from coelomic epithelium, fail to elongate, fuse, or canalize properly [5]. Type 2 MRKH, as in this case, includes renal anomalies such as agenesis or ectopia, reflecting shared embryological origins between Müllerian and Wolffian ducts [6].

Mediastinal Müllerian cysts, also called Hattori cysts, are rare lesions typically found in the posterior mediastinum and exclusively reported in females. They are lined by ciliated tubal-type epithelium expressing oestrogen receptor (ER), progesterone receptor (PR), and PAX8 [4, 7]. Mesothelial cells can exhibit Müllerian differentiation, possibly through metaplastic changes or embryological misplacement [8]. Hattori proposed that these cysts arise from mesothelial cells transforming into Müllerian epithelium [4]. Alternatively, Batt *et al*.[9] linked them to primary Müllerian apparatus remnants, invoking Ludwig’s theory of MRKH pathogenesis, where abnormal duct migration or remnants persist ectopically.

During embryogenesis, the Müllerian ducts form from invaginations of coelomic epithelium around weeks 5–6, growing caudally alongside Wolffian ducts. Disruptions could result in ectopic Müllerian remnants becoming sequestered in cranial locations such as the mediastinum via developmental misplacement [10]. The paravertebral location in our case, adjacent to vertebral bodies, supports this hypothesis [11].

## Conclusion

Surgical resection or de-roofing is the recommended treatment for mediastinal cysts that are symptomatic, enlarging, or indeterminate [12]. VATS is the preferred approach due to its minimally invasive nature and excellent visualization of the posterior mediastinum. Complete excision is ideal for definitive diagnosis and to prevent recurrence; however, if the cyst wall is densely adherent to vital structures such as the spine or aorta, partial resection with cauterization of the remnant is acceptable given the benign nature. No recurrences of mediastinal Müllerian cysts have been reported after surgical removal, and our patient likewise remains disease-free at follow-up.
